# Psychometric properties of the Chinese version of the fathers’ fear of childbirth scale: A cross-sectional study

**DOI:** 10.3389/fpsyt.2023.1128971

**Published:** 2023-02-03

**Authors:** Pingping Guo, Xuehui Zhang, Nianqi Cui, Dandan Chen, Lijuan Wang, Qiong Zheng, Jin Xu, Yin Jin, Minna Mao, Ping Xu, Wei Zhang, Xiaojuan Wang, Xuefen Xu, Rujia Zhao, Suwen Feng

**Affiliations:** ^1^Women’s Hospital, Zhejiang University School of Medicine, Hangzhou, China; ^2^Faculty of Nursing, Zhejiang University School of Medicine, Hangzhou, China; ^3^School of Nursing, Jining Medical University, Jining, China

**Keywords:** fear of childbirth, expectant father, cultural adaptation, psychometric, Chinese

## Abstract

**Background and Aim:**

Fear of childbirth (FOC) is one of the most common mental health concerns among expectant fathers, which can cause adverse consequences for themselves and their families. A valid and accurate tool is the key to the identification of FOC. This study aimed to translate and culturally adapt the fathers’ fear of childbirth scale (FFCS) into simplified Chinese and test the scale’s psychometric properties among expectant fathers in mainland China.

**Methods:**

Researchers obtained translation permission and followed the multiphase translation guidelines to develop the Chinese version of the fathers’ fear of childbirth scale (C-FFCS). Relevant psychometric properties were selected for the scale’s psychometric validation on the basis of the Consensus-based Standards for the Selection of Health Status Measurement Instruments checklist. In this cross-sectional study, two samples of expectant fathers were collected in a university-affiliated hospital in Hangzhou between September and October 2022.

**Results:**

A total of 381 expectant fathers completed the C-FFCS, resulting in an effective response rate of 95.6%. The C-FFCS is a 3-factor structure consisting of 16 items, which explained 66.374% of the total variance. The content validity index of items ranged from 0.833 to 1.00, and the scale-level content validity index was 0.931. The confirmatory factor analysis confirmed the scale’s 3-factor structure. Evidence of convergent validity (average variance extracted = 0.508–0.780) as well as discriminant validity offered excellent psychometric support. The Cronbach’s α coefficient, McDonald’s ω coefficient, intraclass correlation coefficient, Spearman-Brown coefficient, and Guttman split-half coefficient are within the satisfactory range (> 0.80). Significant correlations between the scores of the C-FFCS and Childbirth Attitude Questionnaire (*r* = 0.658, *p* < 0.01) and Fear of Birth Scale (*r* = 0.555, *p* < 0.01) both revealed good concurrent validity. The structure of C-FFCS was invariant across different parity groups, with no floor and ceiling effect.

**Conclusion:**

The C-FFCS was demonstrated to be a sound instrument with good reliability and validity for measuring Chinese expectant fathers’ FOC levels. However, further studies are advocated to verify the C-FFCS among a larger sample that is more representative of the Chinese expectant father population.

## 1. Introduction

According to the International Conference on Population and Development, men’s attendance in all aspects of sexual and reproductive health affairs is crucial for achieving safe motherhood (1). As a matter of fact, many countries, including China, have increasingly encouraged the involvement of the male partner in pregnancy and childbirth during recent decades (1, 2). Such involvement is warmly welcomed by women and regarded as one way of humanizing medicalized birth, which is beneficial to birth outcomes, the father’s own, as well as their families (3). Undoubtedly, expecting childbirth and accepting the role of a father is a valuable and rewarding experience in a man’s life, and is usually considered as a period of self-fulfillment, happiness, belief, and hope (4). Nevertheless, since pregnancy and childbirth is a process filled with uncertainties, multidimensional changes, and potential challenges, it cannot be completely controlled and its outcomes are hard to predict in advance (5), fatherhood can be a difficult phase of transition for a great number of men, even when it is desired and planned for, or in high-income countries where maternity care is often safe (6, 7). Studies (8, 9) on expectant fathers’ experiences have shown that men experience a rising negative emotional response as they take a more active role during pregnancy and childbirth; one of the most common mental health concerns is fear of childbirth (FOC) (10, 11).

Although it is a common clinical problem, no uniform and clear definition for FOC has been settled (12). It has been described in some studies (12, 13) as negative and anxious feelings starting in the antenatal period and mostly experienced during labor, and can be further classified into primary (occurring with no prior childbirth experience) and secondary (occurring following the traumatic experience of previous childbirth) FOC (14). Since the Swedish obstetrician Areskog et al. (15) carried out the first study on FOC in 1981, it has progressively become a widely discussed subject and an increasingly addressed issue both in research and in clinical practice (16, 17). However, pregnant women are usually the focus of research regarding FOC (16), while significantly less attention has been paid to expectant fathers, who are part and parcel of the pregnancy and childbirth process in the broader spectrum of reproductive, maternal, and neonatal health, and may also be very likely to suffer from FOC (6, 18).

Evidence suggests that, similar to pregnant women, expectant fathers’ FOC is also multidimensional, which mainly revolves around the health and well-being of their partner as well as the unborn baby (19). Many other elements that make up FOC in men were also declared, such as fear of inability to cope, not being allowed to participate in important decisions, lack of knowledge, unfamiliar healthcare providers and medical environment (19). In the clinical practice, FOC intensities are commonly classified as mild, moderate, severe, and disabling fear (known as tokophobia) (20). Some level of FOC is regarded as normal and adaptive (21), while its exacerbation during pregnancy and childbirth is undesirable (22). According to the limited existing studies, some degree of FOC is reported by 11–80% of expectant fathers (23, 24), with the prevalence of intense FOC estimates ranging from 5 to 13% (23, 25), depending on the instrument used to measure, chosen cut-off value, populations studied, and the quality of health care provided (12, 17).

Expectant fathers’ FOC can overshadow the entire pregnancy, complicate the childbirth process, and may lead to plenty of negative consequences and outcomes (6, 10). Perhaps most importantly, there was a higher likelihood of subsequent caesarean deliveries as it has been demonstrated that there is a direct and significant relationship between fathers’ FOC and their preference for caesarean delivery (10). More noteworthily, a couple’s preference for the form of childbirth could be influenced by each other (26), which means that a man’s preference for cesarean section may be an important reason both for women to prefer childbirth with cesarean and for the actual caesarean birth (25). Indeed, FOC has been revealed as the root cause of the increased caesarean rates without medical indication (27). According to global statistics (28), caesarean birth rates have been increasing in both developed and developing countries. A study (29) showed that mainland China has the highest caesarean rate (46.2%) in Asia and the maternal requesting caesarean is one of the top contributory factors for this high rate, where it has been growing by 1.0% *per annum* (28). However, the WHO has already stated that caesarean birth rates above 10–15% are clearly associated with more harm than good, which does not help reduce maternal and neonatal mortality rates but can increase medical costs and result in dangerous side effects for the mother and baby (30). Furthermore, expectant fathers with intense FOC are more likely to experience Couvade syndrome than those without or with a low level of FOC, which is manifested as eating disorders, insomnia, chronic fatigue syndrome, headaches, irritability, and erectile problems that closely simulate those of pregnancy and can interfere with work and family activities (18). In addition, other adverse consequences of elevated paternal FOC may include but not be limited to undermining fathers’ capability to support their wives, lowering their self-efficacy to achieve paternal roles, impairing the paternal-infant attachment, jeopardizing the couple relationships, and triggering psychiatric disorders (19, 31). Fortunately, there are opportunities to mitigate these negative effects and provide a more pleasant pregnancy and childbirth experience for the distressed father-to-be by identifying fathers at risk of FOC early and providing them with positive childbirth preparation as well as supportive, appropriate, and timely care (11, 20).

A valid instrument is the key to early detection of FOC and its severity. There are currently numerous tools that could be employed to assess the FOC, including the Childbirth Attitude Questionnaire (CAQ), Fear of Birth Scale (FOBS), Wijma Delivery Expectancy Questionnaire (W-DEQ), Childbirth Fear Questionnaire (CFQ), Fear of Childbirth Questionnaire (FCQ), and Delivery Fear Scale (DFS) (13). Nevertheless, all of these tools were initially developed for use by women. Although some studies (23, 32) have revised these tools by omitting items perceived to be irrelevant to males to improve their utility among men, the accuracy and comprehensiveness of these screening tools for evaluating fathers’ FOC requires further investigation. For the consideration that the content of any instrument needs to be culturally appropriate for the target group for which it is intended (33), Ghaffari et al. (31) developed the first scale (Fathers’ Fear of Childbirth Scale, FFCS) to measure expectant fathers’ FOC levels specifically. The findings of this study (31) suggest that the FFCS possesses good validity and reliability when assessing FOC among Iranian expectant fathers. Nevertheless, given that the existence and adaptation of one scale version does not guarantee measurement equivalence across other populations, whether FFCS is generalizable to different countries and other cultural contexts is still not clear.

To the best of our knowledge, there is no validated instrument specially developed to measure men’s FOC in China. Since assessing the FOC of expectant fathers in China is of paramount importance but developing a new instrument is time-consuming and of high cost, translating and testing a well-validated tool developed in another country is a good alternative. The aims of the present study are to translate the FFCS into Chinese, to conduct its cross-cultural adaptation for Chinese expectant fathers, and to analyze its psychometric properties. We hope that this study serves as a reference for the measurement, assessment, and intervention regimen development of Chinese expectant fathers’ FOC.

## 2. Materials and methods

### 2.1. Study design

A cross-sectional survey study was carried out to evaluate the psychometric properties of C-FFCS. The study followed the guideline for strengthening the reporting of observational studies in epidemiology (STROBE) (34).

### 2.2. Procedures and participants

This study was carried out in the obstetric out-patient clinic of a third-level A-grade obstetrics and gynecology hospital in Zhejiang Province, China from September to October 2022. The eligible criteria of this study were settled by referring to Ghaffari et al.’s study (31) that tested the psychometric properties of the FFCS. Expectant fathers who were age ≥ 18 years old, capable of reading and articulating in Chinese, and who attended the prenatal visits with their spouses in the second or third trimester of pregnancy were included. The exclusion criteria for expectant fathers were: (1) any history of hospitalization in psychiatric hospitals; (2) spouses diagnosed with high-risk pregnancies; and (3) any history of a child with physical or mental abnormalities in the family.

Self-administered pen-and-paper questionnaires consisting of the Chinese version of the FFCS, CAQ, FOBS, and demographic information were distributed to all participants by two well-trained researchers and were retrieved once finished to check the missing content and ensure the questionnaire’s integrity. According to the methodological recommendations for the validation of scales (ten times the number of items of the instrument being validated) (35), a minimum sample size of 170 was estimated ‘*a priori*’. A total of 400 questionnaires were eventually distributed because of the sufficient sample sources, and 381 were usable. To elaborate a structure for the questionnaire, the dataset was randomly split into two halves to perform exploratory factor analysis (EFA; sample 1: *N* = 170) and confirmatory factor analysis (sample 2: *N* = 211).

### 2.3. Instruments

#### 2.3.1. Demographic characteristic

A self-compiled basic information form was used to capture the characteristics of participants, including age, education, marital status, employment status, family income, gestational age, parity, and so on.

#### 2.3.2. Fathers’ fear of childbirth scale

The FFCS is a self-reported instrument initially constructed by Ghaffari et al. (31) to investigate the levels of childbirth fear among expectant fathers, which is composed of 17 items and divided into 2 domains entitled “fear of childbirth” and “fear of hospital.” Each item of FFCS is rated on a 5-point Likert scale: 1 = “do not agree at all,” 2 = “do not agree,” 3 = “no idea,” 4 = “completely agree,” and 5 = “completely agree.” The score range of the original version of the FFCS is between 17 and 85, and higher scores indicate more severe childbirth fear. The initial psychometric evaluation of FFCS revealed good face validity, content validity, structure validity, and internal consistency (Cronbach’s *α* = 0.84; 31).

#### 2.3.3. Childbirth attitude questionnaire

The CAQ was developed by Hartmann to measure childbirth fear (36). The adapted Chinese version of the CAQ consists of 16 items and 4 domains (37): fear of fetal health; fear of childbirth pain; fear of losing control during childbirth; and fear of medical intervention as well as the hospital environment. Item responses of the CAQ are based on a 4-point Likert scale (1–4) with a possible total score ranging from 16 to 64. Higher scores represent a more severe level of childbirth fear. The reliability and validity of the Chinese version of the CAQ have been extensively verified (37, 38). For this study, the Cronbach’s α coefficients of the CAQ and each subscale were 0.933, 0.820, 0.846, 0.819, and 0.692, respectively. The CAQ was used for testing concurrent validity.

#### 2.3.4. Fear of birth scale

The FOBS was initially developed by Haines et al. (39) and tested among pregnant women to measure the fear of childbirth and has subsequently also been applied to expectant fathers (40). On this scale, the participants will be required to rate their feelings in response to the question, “How do you feel right now about the approaching birth?” by marking the statements (i) “calm/worried” and (ii) “no fear/strong fear” on two 100-mm visual analog scales, which are summed and averaged to get a final score. The total score of FOBS ranges from 0 to 100, with higher scores representing a higher level of fear of birth. The FOBS is freely available for academic purposes (40). In this study, FOBS was first translated into the Chinese language by using the same translation and cross-cultural adaptation method as for the FFCS. The Cronbach’s α (0.938) and ICC (0.893) of FOBS in this study were excellent. The FOBS was also used for testing concurrent validity.

### 2.4. Study phase I: Instrument construction

The first author of this study obtained official permission from the main developer (Dr. Shahhosseini) of the FFCS to translate the English version of the FFCS into the Chinese language. The translation and cross-cultural adaptation procedures for the FFCS were performed on the basis of international guidelines (41). The detailed steps included i—forward translation and synthesis; ii—back translation; iii—specialist committee review; iv—external expert consultation for content validity; and v—pilot testing.

Step i: Two independent native Chinese translators (one was a translation expert and the other had a medical background) translated the original questionnaire from English into the simplified Chinese version, with a conceptual translation being preferred over a literal one. After addressing any divergence about the words, phrases, and items, a consensus was reached and the synthesis forward translation version was obtained.

Step ii: A back translation with the synthesis translated version was made by two other bilingual (English/Chinese) translators independently and created two back-translations. Both translators were blind to the original version and had no medical background.

Step iii: A specialist committee was set up, which was comprised of the four translators, three obstetrics experts, one psychologist, one methodologist, and one linguistics professional. The specialist committee reviewed all reports, discussed any issues, and compared the reports with the original version of FFCS to reach a consensus on discrepancies and achieve semantic, idiomatic, experiential, and conceptual equivalence. We sent the synthesis forward translation version and two back-translated-English versions to Dr. Shahhosseini and integrated his feedback to produce a Chinese consensus version and a back-translated English consensus version.

Step iv: External expert surveys were carried out to determine the content validity of the Chinese consensus version by assessing each item on a 4-point ordinal rating scale (4 = Very relevant and succinct; 3 = The item should be revised; 2 = The item should be seriously revised, 1 = Not relevant) to confirm whether the content of an instrument adequately reflects the construct to be measured. According to an internationally accepted guideline (41), we emailed the survey to six experts (an obstetric physician, a mental health expert, two obstetric nursing specialists, and two nursing professors) and got feedback from all of them in both rounds of surveys. The specialist committee made some necessary revisions based on the external experts’ suggestions to achieve the final harmonized preliminary Chinese version (C-FFCS-FHP) for pilot testing.

Step v: To verify comprehension of the meaning and wording of test items, i.e., face validity, the C-FFCS-FHP was administered to a purposive sample of 30 eligible expectant fathers with diverse characteristics in terms of age, education, and occupational background (41). Meanwhile, the time needed to complete the questionnaire was documented. Then, the specialist committee fine-tuned the C-FFCS-FHP based on the outcome of the pilot testing. Dr. Shahhosseini further approved the refined C-FFCS-FHP that was ready to undergo field testing for the validation of psychometric properties.

### 2.5. Study phase II: Instrument validation

According to the recommendation in the Consensus-based Standards for the Selection of Health Status Measurement Instruments (COSMIN) checklist (42), we evaluated the psychometric properties of the C-FFCS, including item analysis, structural validity, convergent and discriminant validity, measurement invariance, reliability, floor/ceiling effects, and concurrent validity.

### 2.6. Data analysis

The Statistical Package for Social Sciences Version 25.0 for Windows was used for most calculations. CFA and multigroup CFA were conducted *via* AMOS (version 24, IBM, Chicago, IL, United States), and McDonald’s ω was calculated *via* jamovi V.2.2.5. Descriptive statistics [i.e., frequencies and percentages (%), and means ± standard deviations (SD)] were determined to characterize the demographic data of the sample. A value of *p* of < 0.05 was deemed statistically significant.

#### 2.6.1. Content validity

Content validity was assessed with the content validity index (CVI), both at the item level (I-CVI) and the scale level (S-CVI). The I-CVI is the proportion of experts with scores ≥ 3 on a 4-point scale. The S-CVI was obtained by calculating the mean CVI values. I-CVI ≥ 0.78 and S-CVI ≥ 0.8 is generally regarded as excellent when there are six or more experts rating the items (43).

#### 2.6.2. Item analysis

Item analysis was conducted based on the following analyses (44–46): (1) extreme group comparison: the upper 27% and lower 27% scoring groups of items should be significantly discriminated; (2) critical ratio (CR): items with a CR ≥ 3.0 and a *p* value < 0.05 were retained; (3) correlation coefficient method: items that had an item-total correlation < 0.30 were excluded; and (4) Cronbach’s *α* or McDonald’s *ω* significantly improved (an increase of 0.5 or more in the alpha or *ω* coefficient for the overall scale) if an item was deleted.

#### 2.6.3. Structure validity

The factor structure of C-FFCS was examined using EFA and CFA. Prior to EFA, the suitability of the study data for factor analysis was confirmed using the Kaiser–Meyer–Olkin (KMO) test, with > 0.6 considered acceptable and a significant Bartlett’s test of sphericity (44, 45). The principal component analysis and oblique rotation (promax criterion) were used in EFA for the extraction of factors as the correlation coefficient between the factors was greater than 0.3 (45). To identify the number of factors to be retained, the following criteria were applied (45, 46): (1) eigenvalues above 1; (2) factor loading of ≥ 0.4; (3) theoretical considerations; and (4) Cattell’s scree plot. Items were omitted one at a time, reperforming the analysis at each step to achieve an optimal solution (47). For CFA, a maximum-likelihood (ML) estimator was utilized to estimate the parameters. When commenting on the fit of the CFA model, the following indices of goodness-of-fit were considered: chi-square/degree of freedom (*χ*^2^/*df*), comparative fit index (CFI), goodness of fit (GFI), Tucker–Lewis index (TLI), incremental fit index (IFI), and root mean square error of approximation (RMSEA). The model fit was considered to be acceptable with CFI, GFI, TLI, and IFI ≥ 0.80, and RMSEA ≤ 0.08, whereas the model fit was regarded as excellent when CFI, GFI, TLI, and IFI ≥ 0.90, and RMSEA ≤ 0.05 (45, 46).

#### 2.6.4. Convergent and discriminant validity

Internal convergent validity of factors was evaluated by average variance extracted (AVE), with scores ≥ 0.5 denoting satisfactory convergent validity (48). To demonstrate discriminant validity, the √AVE score should exceed each of its correlations with other factors (48, 49).

#### 2.6.5. Measurement invariance

Cross-cultural validity is needed to assess measurement invariance when an instrument is applied to different “cultural” groups (42), such as groups of different demographic and clinical characteristics. We used multigroup CFA to examine whether factor structure and model fit are acceptable across different parity groups, namely configural invariance, to test the cross-cultural validity of the C-FFCS. The sample sizes of each group met the criteria of more than 5 times the number of items and ≥ 100 (50).

#### 2.6.6. Reliability

Cronbach’s *α* was calculated for internal consistency analysis. However, given the assumptions of Cronbach’s *α* being frequently violated in psychology, internal consistency was also assessed using McDonald’s *ω* (51). Adequate internal consistency was defined by a Cronbach’s *α* as well as McDonald’s *ω* of > 0.7 (51). The Spearman-Brown coefficient and the Guttman split-half coefficient were used to test the split-half reliability of the scale. An index higher than 0.7 indicates optimal stability (45). Additionally, the test–retest reliability was estimated by the intraclass correlation coefficient (ICC). Thirty-six expectant fathers were completed with the C-FFCS at a 14-day interval to establish test–retest reliability since the assessment scores were relatively stable over 1–2 weeks (52). ICC values of 0.75–1.0 were considered excellent agreement (53).

#### 2.6.7. Floor/ceiling effect

To assess interpretability, the floor/ceiling effect [more than 15% of responses in the lowest or highest option (42)] for the total scale was examined.

#### 2.6.8. Concurrent validity

Concurrent validity was assessed using Spearman’s correlation coefficients between the final C-FFCS and other instruments, including the CAQ and FOBS. Concurrent validity was recognized if the correlation coefficient |r| is 0.45 or higher (54).

### 2.7. Ethics consideration

The study was approved by the ethics committee of the Institutional Review Board of the hospital (IRB-20220242-R). All procedures complied with the ethical guidelines of the World Medical Association’s Helsinki declaration (55). All participants signed an informed consent form and were aware of the right to withdraw their consent at any stage without any penalty. There was no financial compensation for respondents. The survey did not disclose any personal information, and all collected data remained confidential.

## 3. Results

### 3.1. Sample characteristics

Overall, 381 out of the 400 participants completed the survey, with an effective response rate of 95.25%. The mean age of samples in the study was 31.99 ± 3.94. Expectant fathers who were employed, married, lived in urban areas, at the third trimester of pregnancy (≥ 28 weeks), and in their spouses’ first pregnancy were in the majority. The introductory characteristics of the participants are reported in Table 1.

**Table 1:** Characteristics of the included participants (*N* = 381).

Characteristics	Mean (standard deviation)/*N* (%)		
	Total Sample *N* = 381	Sample 1 *N* = 170	Sample 2 *N* = 211
*Age, years*	31.99 ± 3.94	31.70 ± 3.65	32.23 ± 4.15
*Education*			
High school/specialized secondary school or below	36	17	19
Specialty/Bachelor	264	121	143
Postgraduate or above	81	32	49
*Marital status*			
Married	376	169	207
Unmarried	5	1	4
*Residence*			
Urban	324	144	180
Rural	57	26	31
*Household monthly income (Chinese Yuan)*			
≤10,000	77	39	38
10,001–20,000	125	53	72
> 20,000	179	78	101
*Employment status*			
Employed	372	164	208
Unemployed	9	6	3
*Gestational age*			
12–27^+6^ weeks	91	46	45
≥28 weeks	290	124	166
*Parity*			
Primiparity	281	135	146
Multiparity	100	35	65
*Pregnancy planning*			
Yes	320	146	174
No	61	24	37
*Expected mode of delivery*			
Vaginal delivery	305	137	168
Cesarean section	76	33	43
*Use of the pregnancy-related APPs*			
Yes	279	132	147
No	102	38	64

### 3.2. Cross-cultural adaptation, face validity, and content validity

Some items were modified slightly in the development of C-FFCS. For instance, the “time of childbirth” was changed to the “due date of childbirth” in item 1; “dangerous medical interventions” in item 8 of the original scale was adjusted to “extra medical interventions” in order to avoid expectant fathers’ antipathy; the “pain” was crystallized into “childbirth pain” in item 9; the phrase “hospital staff will not have enough skills” in item 15 was extended to “hospital staff will not have enough skills or not be careful enough” based on the feedback from pilot testing; and item 7 “I’m afraid that my spouse’s childbirth will be risky” and item 11 “I am afraid that my child’s health will be endangered due to childbirth” were revised to “I am afraid that my spouse will have an accident during childbirth” and “I am afraid that my child will have an accident during childbirth” respectively, according to Chinese reading and expression habits. The results of the quantitative content validity assessment in the second round of external expert consultations indicated that the S-CVI was 0.931, and the I-CVI ranged from 0.833 to 1.00. To assess the qualitative face validity, the C-FFCS-FHP was pre-tested among 30 expectant fathers to understand how they perceived and interpreted the items. The participants stated that most of the wording of C-FFCS-FHP (see Supplementary Table S1 for details) was clear and easy to understand. It takes about 3–5 min to fill out the scale. Ultimately, none of the items from the original FFCS were deleted, and all of the 17 items in C-FFCS-FHP were used for the next phase of psychometric validation.

### 3.3. Psychometric analysis

#### 3.3.1. Item analysis

The extreme groups’ comparison (Table 2) showed that the differences between the top and bottom 27% of all items reached significance (*p* < 0.01), and the CR value of all items exceeded 3.0. Then, the Spearman’s correlation method revealed that each item-total correlation was positive, with all coefficients of items being > 0.3 except for item 3 (0.283). However, given that the coefficient value of item 3 was very close to 0.3, it was temporarily retained. Moreover, there was no sign of growth both in α or ω coefficient by removing any item. Therefore, no items were deleted in the item analysis.

**Table 2:** Item analysis (*N* = 170).

Item	Extreme group comparison	Item-total correlation	Cronbach’s α if item deleted	McDonald’s omega if the item is deleted	Numbers of substandard indicators	Note
	Criterial ratio					
1	6.497^**^	0.563^**^	0.916	0.916	0	Retained
2	7.612^**^	0.542^**^	0.916	0.916	0	Retained
3	3.767^**^	**0.283** ^**^	0.922	0.923	1	Retained
4	10.576^**^	0.701^**^	0.912	0.912	0	Retained
5	11.907^**^	0.705^**^	0.912	0.912	0	Retained
6	8.846^**^	0.629^**^	0.914	0.915	0	Retained
7	12.077^**^	0.761^**^	0.911	0.911	0	Retained
8	7.515^**^	0.584^**^	0.916	0.916	0	Retained
9	7.169^**^	0.600^**^	0.915	0.915	0	Retained
10	7.436^**^	0.574^**^	0.915	0.915	0	Retained
11	11.871^**^	0.745^**^	0.910	0.911	0	Retained
12	11.019^**^	0.716^**^	0.911	0.911	0	Retained
13	8.717^**^	0.567^**^	0.914	0.915	0	Retained
14	10.548^**^	0.632^**^	0.914	0.915	0	Retained
15	10.382^**^	0.632^**^	0.914	0.914	0	Retained
16	11.551^**^	0.686^**^	0.912	0.913	0	Retained
17	12.953^**^	0.707^**^	0.912	0.912	0	Retained

Bold values is the character that mean the indicator is substandard. C-FFCS, Chinese version of the fathers’ fear of childbirth scale. ^**^p < 0.01.

#### 3.3.2. Structure validity

**Figure 1:**
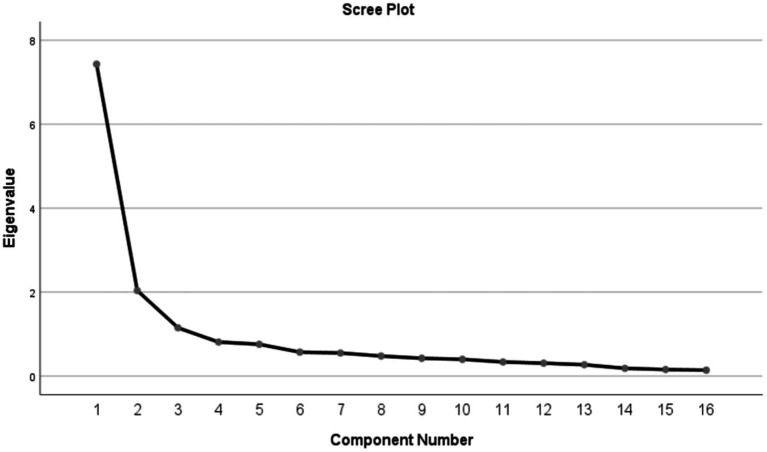
The scree plot.

We conducted two EFAs on the C-FFCS-FHP. Before EFA, the KMO index of 0.900 and Bartlett’s test of sphericity (*χ*^2^ = 1694.040, *df* = 120, *p* < 0.000) confirmed the data adequacy for factor analysis. In the primary EFA of 17 items, item 3 was deleted for the reason that it did not load on any factor. In the second EFA of the remaining 16 items, three factors with eigenvalues > 1.0 (7.431, 2.038, and 1.151) were extracted, which explained 66.374% of the total variance (Table 3). Factor loadings of items were determined to range from 0.450 to 0.943 (see pattern matrix in Table 3). The scree plot further confirmed the 3-factor structure (Figure 1).

**Table 3:** The exploratory factor analysis results of the C-FFCS (*N* = 170).

Item number and description	Pattern Matrix			Structure Matrix		
	Factor 1	Factor 2	Factor 3	Factor 1	Factor 2	Factor 3
*Factor 1*						
Item 11	**0.876**	0.012	−0.005	0.878	0.429	0.550
Item 10	**0.816**	−0.083	−0.031	0.757	0.296	0.448
Item 7	**0.781**	−0.025	0.103	0.834	0.391	0.583
Item 17	**0.671**	0.319	−0.127	0.744	0.589	0.424
Item 8	**0.656**	0.152	−0.121	0.652	0.417	0.352
Item 9	**0.654**	−0.090	0.142	0.700	0.281	0.516
Item 1	**0.450**	−0.154	0.384	0.617	0.217	0.604
*Factor 2*						
Item 14	−0.095	**0.943**	0.057	0.393	0.921	0.379
Item 15	0.054	**0.926**	−0.078	0.449	0.921	0.331
Item 16	0.078	**0.837**	0.016	0.489	0.880	0.404
Item 13	−0.044	**0.819**	0.091	0.405	0.835	0.395
*Factor 3*						
Item 2	−0.073	−0.113	**0.886**	0.429	0.210	0.795
Item 5	−0.088	0.110	**0.882**	0.519	0.425	0.871
Item 6	−0.066	0.164	**0.718**	0.463	0.423	0.743
Item 4	0.292	−0.075	**0.632**	0.653	0.321	0.785
Item 12	0.172	0.142	**0.620**	0.629	0.475	0.785
% of the variance	46.443	12.74	7.191	**–**	**–**	**–**
Cumulative variance	46.443	59.182	66.374	**–**	**–**	**–**

C-FFCS, Chinese version of the fathers’ fear of childbirth scale; Factor loadings with an absolute value > 0.40 are displayed in bold in the Pattern Matrix.

**Figure 2:**
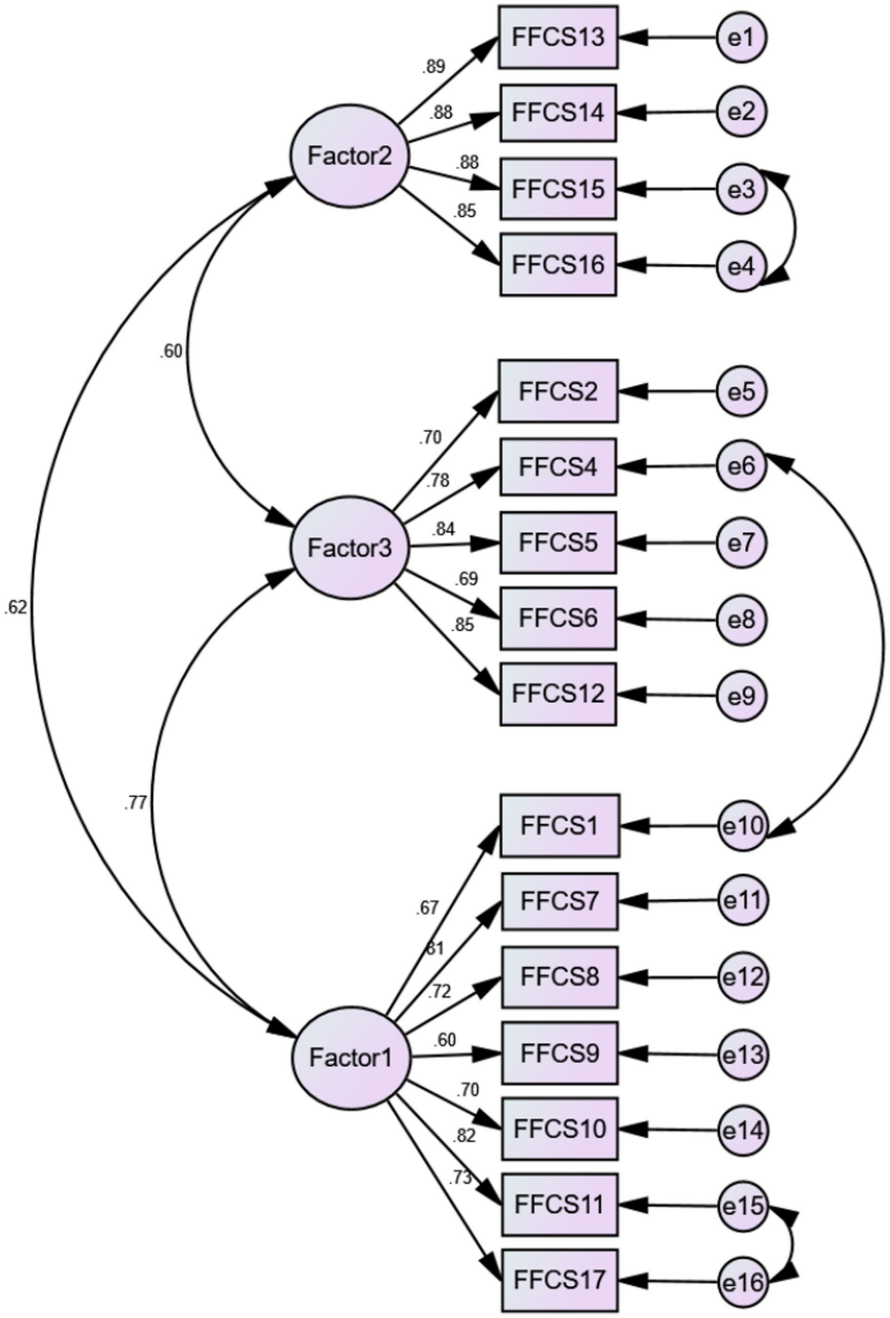
Modified confirmatory factor analysis of the three factor 16-item model.

Confirmatory factor analysis was carried to verify the EFA-generated 16-item 3-factor structure. The initial model indices showed an inadequate fit based on the GFI, IFI, CFI, and TLI (*χ*^2^/df = 3.509, RMSEA = 0.109, GFI = 0.826, IFI = 0.891, CFI = 0.890, and TLI = 0.869). When respecifying the model by including three error covariances according to the modification indices, the model fit improved greatly: *χ*^2^/*df* = 2.480, RMSEA = 0.084, GFI = 0.882, IFI = 0.938, CFI = 0.937, and TLI = 0.923 (Figure 2; Table 4).

**Table 4:** Results of the confirmatory factor analysis of the C-FFCS (*N* = 211).

Model fit indices	*χ*^2^/*df*	CFI	GFI	TLI	IFI	RMSEA
Standard model fit	3.509	0.890	0.826	0.869	0.891	0.109
Adjusted model fit	2.480	0.937	0.882	0.923	0.938	0.084

C-FFCS, Chinese version of the fathers’ fear of childbirth scale; *χ*^2^/*df*, Chi-square/degree of freedom; CFI, comparative fit index; GFI, goodness of fit; TLI, Tucker–Lewis index; IFI, incremental fit index; RMSEA, root mean square error of approximation.

#### 3.3.3. Convergent and discriminant validity

Good convergent validity was found because of each AVE exceeding 0.50. In addition, all subscales’ √AVE scores exceeded each of their correlations with other subscales, revealing satisfactory discriminant validity (Table 5).

**Table 5:** Correlation coefficient and AVE (*N* = 170).

C-FFCS	AVE	Inter-dimension correlations		
		1	2	3
Factor 1	0.508	**0.713**		
Factor 2	0.780	0.468^**^	**0.883**	
Factor 3	0.572	0.649^**^	0.413^**^	**0.756**

AVE, average variance extracted; C-FFCS, Chinese version of the fathers’ fear of childbirth scale; ^**^p < 0.001; ^*^p < 0.05. Bold characters show the square of root of average variance extracted values.

#### 3.3.4. Measurement invariance

The multiple-group CFA with the unconstrained model revealed an acceptable baseline model for both primiparity and multiparity expectant fathers (*χ*^2^/*df* = 2.077, IFI = 0.946, TLI = 0.934, CFI = 0.946, RMSEA = 0.053), demonstrating no parity differences.

#### 3.3.5. Reliability

The Cronbach’s *α*, McDonald’s *ω*, as well as the ICC of the C-FFCS and the three factors shown in Table 6 with all values > 0.8, demonstrate excellent reliability. The Spearman-Brown coefficient and Guttman split-half coefficient of the C-FFCS were both 0.860.

**Table 6:** Reliability analysis of the C-FFCS (*N* = 381).

	C-FFCS	Factor 1	Factor 2	Factor 3
Cronbach’s α coefficient	0.922	0.862	0.874	0.876
McDonald’s omega coefficient	0.925	0.870	0.880	0.878
Intraclass correlation coefficient	0.818	0.931	0.814	0.927

C-FFCS, Chinese version of the fathers’ fear of childbirth scale.

#### 3.3.6. Floor/ceiling effect

No ceiling or floor effects were detected in the current study because less than 15% of expectant fathers achieved the highest or lowest score in the C-FFCS and three dimensions.

#### 3.3.7. Concurrent validity

The correlation of the C-FFCS with the CAQ was 0.658 and with the FOBS was 0.555 (*p* < 0.01; Table 7 shows this in more detail), demonstrating good concurrent validity.

**Table 7:** Correlations of the C-FFCS, CAQ, and FOBS (*N* = 381).

	C-FFCS	Factor 1	Factor 2	Factor 3
CAQ	0.658^**^	0.615^**^	0.526^**^	0.527^**^
Subscale 1	0.595^**^	0.590^**^	0.435^**^	0.453^**^
Subscale 2	0.612^**^	0.592^**^	0.469^**^	0.485^**^
Subscale 3	0.595^**^	0.510^**^	0.502^**^	0.520^**^
Subscale 4	0.648^**^	0.441^**^	0.892^**^	0.433^**^
FOBS	0.555^**^	0.484^**^	0.384^**^	0.547^**^

C-FFCS, Chinese version of the fathers’ fear of childbirth scale; CAQ, childbirth attitude questionnaire; FOBS, fear of birth scale; ^**^p < 0.001.

### 3.4. Scale summary

The final version of the C-FFCS has 16 items grouped into 3 subscales (Supplementary Table S1). Specifically, subscale 1 included seven items related to “fear of the health and safety of mother and baby” (items 1, 7, 8, 9, 10, 11, and 17), subscale 2 involved four items related to “fear of the quality of medical care services” (items 13–16), and subscale 3 contained five items related to “fear induced by individual factors” (items 2, 4, 5, 6, and 12). All items are forward-scored using a 5-point Likert response format from 1 (I do not agree at all) to 5 (I completely agree). The total score of the C-FFCS ranged from 16 to 80 with higher scores denoting more severe childbirth fear level.

## 4. Discussion

This study aimed to translate and culturally adapt the FFCS into simplified Chinese and to assess the psychometric properties of the scale following the COSMIN checklist (42). As far as we know, the resulting C-FFCS is the first instrument specifically for evaluating the FOC of Chinese expectant fathers. Results of the psychometric validation revealed excellent internal consistency reliability, sufficient stability (ICC = 0.814–0.931), acceptable validity (content validity, structural validity, convergent and discriminant validity, measurement invariance, and concurrent validity), and no floor/ceiling effect. The 3-factor and 16-item construct of C-FFCS explained 66.374% of the total variance. Overall, C-FFCS can be recommended as an appropriate tool for measuring the FOC among expectant fathers in mainland China.

The international multiphase translation guidelines (41) were strictly followed during the translation and cross-cultural adaptation to improve the standardization and scientificity of the C-FFCS development process. We also solicited and integrated opinion from Dr. Shahhosseini, the developer of the FFCS, and extensively discussed and reconciled the revision contents in the specialist committee to ensure the equivalence of content and structure of the scale. Actually, most items appeared to have culturally equivalent terms in simplified Chinese, so we were able to translate the FFCS without extensive adaptation. On the contrary, minor adjustments and modifications were made in the localization process to make the C-FFCS more comprehensive and explicit, and more understandable and acceptable to Chinese expectant fathers. Eventually, the C-FFCS showed sufficient content validity in the second round of expert consultations (I-CVI = 0.833–1.00 and S-CVI = 0.931), and participants in the pilot testing stated that the scale was easy to understand and the questions aligned well with their feelings, with only 3–5 min to complete.

The KMO test and Bartlett spherical test were carried out beforehand to settle the suitability of factor analysis, which aims to determine the degree to which sample scores on a tool can reflect the dimensionality of the structure being assessed (45). The results of the KMO value of 0.900 and Bartlett spherical test (*χ*^2^ = 1694.040, *df* = 120, *p* < 0.000) confirmed this suitability. In EFA, item 3 (“I worry that the quality of sex with my spouse will decline after childbirth.”) was deleted due to only one item in a different dimension of content (45). This deletion corroborated the results of the item analysis, as item 3 was the only one of all the items that had substandard indicators in the item analysis, despite it being temporarily retained, considering that the item-total correlation coefficient of 0.283 for item 3 was very close to the criterion of 0.3. The reason for the misfit of item 3 may be related to different cultural contexts and socially constructed norms. Sex life has always been considered an extremely private and non-disclosed topic in China, for which Chinese people may not want to be aware of their attention being drawn to this topic by others and can be reluctant to talk about it (56). Moreover, the worry about the quality of sex after childbirth may pale in comparison to other aspects of FOC (24). After omitting the item 3, the second EFA determined 3-factor structure of C-FFCS, unlike the 2-factor structure of the original scale. The factors were given appropriate labels based on the evidence from existing literature and incorporating feedback from experts and the developer of the original scale: fear of the health and safety of mother and baby (Factor 1), fear of the quality of medical care services (Factor 2), and fear induced by individual factors (Factor 3). More than 66% of the total variance could be accounted for by the 3-factor model, better than the 50.82% shown in the original version (31), which signified that the C-FFCS was more in line with the Chinese cultural background after adjustment. Especially to deserve to be mentioned, the maximum expressed variations (46.443%) were linked to Factor 1, revealing that the main concern described by expectant fathers was for the health and safety of their partner and baby. This finding is consistent with previous studies (19, 57), which also reported that the most frequent fear mentioned was the fear of mother’s and baby’s health. It enlightens healthcare professionals that they are quite necessary to provide adequate education and information to expectant fathers to inform them of effective ways of improving the health and safety of mothers and babies during pregnancy and childbirth, thereby reducing the FOC contrapuntally.

The compatibility of sample data with the EFA-derived 3-factor model was estimated by CFA. Since the performance of the fit indices of the initial CFA model was less satisfactory, we conducted the incorporation of modification indices to improve the goodness of fit on the basis of modification recommendations. Finally, three error covariances of 6 items were added (Figure 2), because the results of the initial CFA model showed certain correlations between the above items, and after reading the corresponding items carefully, we believe that there are indeed strong correlations between these items and that these correlations are plausible from a clinical point of view. Concretely, item 1 and 4 are both related to the spouse’s childbirth process; item 11 and 17 are regarding the baby’s safety; whereas item 15 and 16 are about medical staff and medical facilities. Ultimately, the modified model showed an admissible fit, which suggested that the model is capable of sufficiently explaining the desired structure. Furthermore, satisfactory convergent (AVE = 0.508, 0.780, 0.572) and discriminant validity supported the reasonable fit of the 3-factor model as well. Additionally, no cross-parity differences were observed regarding the C-FFCS in the subgroups of primiparity and multiparity expectant fathers, which meant that the scale is measurement invariant and effective between parity groups. In summary, C-FFCS can be considered as a potentially helpful instrument to measure expectant fathers’ FOC in mainland China.

In terms of reliability, the internal consistency Cronbach’s α of C-FFCS and its subscales (0.862–0.922) are in parallel to the results of the original version of FFCS (*α* = 0.861–0.908) (31), which are much higher than the recommended Cronbach’s *α* of ≥ 0.70 (51), indicating that the FFCS has good internal consistency when used with different populations. A more accurate internal consistency coefficient, McDonald’s *ω* also exhibited good reliability with an overall McDonald’s *ω* of 0.925 and for each subscale (0.870, 0.880, and 0.878), and further demonstrated that all of the items contribute to the global construct measured. What is more, the ICC values (0.814–0.931) that were calculated for the first time indicated a high level of stability over time for the C-FFCS. This time-invariant feature of C-FFCS is of great importance when assessing the effectiveness of interventions for alleviating expectant fathers’ FOC. In addition, a dearth of the floor/ceiling effect for the C-FFCS total score suggested that the C-FFCS is capable of discriminating between responders at either extreme of the scale, which supported its applicability to Chinese expectant fathers.

In this study, the concurrent validity analysis that was absent in the sample of Iranian expectant fathers (31) added another source of evidence to support the validity of the C-FFCS. Two scales were selected for concurrent validity evaluation. Specifically, the CAQ is the most widely used tool for measuring FOC in mainland China (37, 38), whereas the FOBS is a two-item visual analogue scale which is quick and easy to finish (40). As expected, C-FFCS was significantly correlated to the CAQ (*r* = 0.433–0.658, *p* < 0.01) and FOBS (*r* = 0.384–0.555, *p* < 0.01), respectively. Hence, we conclude that the C-FFCS was sensitive enough to assess the similar feature as the CAQ and the FOBS. Noteworthily, the W-DEQ is regarded as the gold standard for evaluating FOC to some extent (13), but the reliability and validity of this scale is unknown among expectant fathers in mainland China. Hence, further studies should test the psychometric properties of the W-DEQ rigorously in expectant fathers on China’s mainland and calculate the bivariate correlation between the W-DEQ and the C-FFCS to provide additional important information on the concurrent validity of the C-FFCS.

However, the present study is not exempt from limitations. First, the work was undertaken in a university-affiliated hospital in a large urban area, and most of the participants were well-educated and had a monthly household income higher than the national average, warranting sample homogeneity but limiting the generalizability of the findings. It will be useful for future studies to utilize population-based systematic recruitment strategies to include diverse groups of expectant fathers in a more representative manner, for example, by recruiting more samples from rural areas to establish the Chinese norm of the C-FFCS. Second, the psychometric validation in the current study was based on the classic theory test. Future research may add important information on C-FFCS psychometric properties based on the item response theory using Rasch analysis techniques (58). Third, a social desirability bias and inaccurate reporting might have occurred since the findings of this study depended exclusively on the self-reported data collection method, especially for the consideration of men’s general reluctance to express their feelings due to the masculine norm of self-reliance and the expectation of non-emotional men (59). It will be beneficial to combine qualitative research methods in the future to better capture the complex experience and provide more insight into the origin or cause of expectant fathers’ FOC that the scale-based method failed to assess. Fourth, the lack of research that translated and validated the FFCS in other cultures makes it more difficult to discuss and compare the results of our study. To widely use this scale, additional studies need to be carried out in other countries with different cultures.

The C-FFCS has several potential academic and clinical implications. On the individual level, the C-FFCS scores can serve as an opportunity to start a disclosure discussion about the expectant fathers’ FOC and other mental concerns during pregnancy and childbirth, which are usually considered unspeakable and are hidden as a result of the unconscious stereotyped image of “maleness” in our society (59, 60), i.e., being strong and self-confident. At the academic level, C-FFCS can pave the way for other researchers to further perform research related to expectant fathers’ FOC and its influencing factors. At the clinical practice level, healthcare professionals can use C-FFCS to identify expectant fathers with high FOC as well as the areas to focus on clinical practice and to tailor appropriate interventions with individual men to decrease the risk of future negative consequences, both psychological and obstetric. Moreover, the scale will also allow the detection of changes within an individual, thus aiding the efficient evaluation of programs that aim to improve FOC and facilitating the adjustment of the treatment approach as required. On the global level, on the one hand, our findings provided the world with Chinese data on the expectant fathers’ FOC, enriching the evidence in this research area; and on the other hand, the translation and adaptation of an instrument into different languages will permit the instrument to be utilized in comparative international multicenter studies. Nevertheless, the psychometric properties of the FFCS should be investigated across more cultures to further confirm its robustness as an assessment tool.

## 5. Conclusion

Collectively, the 3-dimension and 16-item C-FFCS has demonstrated satisfactory reliability and validity. Health professionals can use it to measure mainland Chinese expectant fathers’ FOC, thereby developing targeted FOC reduction regimens to improve the intervention efficiency. However, the scale should be further verified in a larger sample that is more representative of the Chinese expectant father before widespread use.

## Data availability statement

The raw data supporting the conclusions of this article will be made available by the authors, without undue reservation.

## Ethics statement

The studies involving human participants were reviewed and approved by Institutional Review Board of Women’s Hospital, Zhejiang University School of Medicine. The patients/participants provided their written informed consent to participate in this study.

## Author contributions

PG, XZ, NC, DC, LW, QZ, JX, YJ, MM, PX, WZ, XW, XX, RZ, and SF: made substantial contributions to conception and design, or acquisition of data, or analysis and interpretation of data; involved in drafting the manuscript or revising it critically for important intellectual content, given final approval of the revision to be published. Each author should have participated sufficiently in the work to take public responsibility for appropriate portions of the content; agreed to be accountable for all aspects of the work in ensuring that questions related to the accuracy or integrity of any part if the work are appropriately investigated and resolved. All authors contributed to the article and approved the submitted version.

## Funding

This study was supported by research grants from the Zhejiang Province Medicine and hygiene Technology Project (2023KY126) in 2023 and the China Scholarship Council.

## Conflict of interest

The authors declare that the research was conducted in the absence of any commercial or financial relationships that could be construed as a potential conflict of interest.

## Publisher’s note

All claims expressed in this article are solely those of the authors and do not necessarily represent those of their affiliated organizations, or those of the publisher, the editors and the reviewers. Any product that may be evaluated in this article, or claim that may be made by its manufacturer, is not guaranteed or endorsed by the publisher.

## Abbreviations

AVE: average variance extracted
CAQ: Childbirth Attitude Questionnaire.
CFA: confirmatory factor analysis.
CFI: comparative fit index.
CFQ: Childbirth Fear Questionnaire.
C-FFCS: Chinese version of the fathers’ fear of childbirth scale.
C-FFCS-FHP: final harmonized preliminary Chinese version of the fathers’ fear of childbirth scale.
COSMIN: consensus-based standards for the selection of health status measurement instruments.
CR: critical ratio.
CVI: content validity index.
DFS: delivery fear scale.
EFA: exploratory factor analysis.
FCQ: fear of childbirth questionnaire.
FFCS: fathers’ fear of childbirth scale.
FOBS: fear of birth scale.
FOC: fear of childbirth.
GFI: goodness of fit.
ICC: intraclass correlation coefficient.
I-CVI: content validity index of items.
IFI: incremental fit index.
KMO: Kaiser-Meyer-Olkin.
RMSEA: root mean square error of approximation.
S-CVI: scale-level content validity index.
SD: standard deviations.
STROBE: the guideline for strengthening the reporting of observational studies in epidemiology.
TLI: Tucker–Lewis index.
W-DEQ: Wijma delivery expectancy questionnaire
