# Supplementary material for: Psychometric properties of the Chinese version of the fathers’ fear of childbirth scale: A cross-sectional study
Source: Front Psychiatry. 2023 Feb 3;14:1128971. doi: 10.3389/fpsyt.2023.1128971 (PMC9935613; doi:10.3389/fpsyt.2023.1128971)
Supplement: Supplementary file 1 [file Table_1.DOCX]

Supplementary Material

# Supplementary Tables

**Table S1**. Items of the origin FFCS, C-FFCS-FHP, and final C-FFCS.

| Item number | Original version of the FFCS | C-FFCS-FHP | Description of the C-FFCS-FHP | C-FFCS |
| --- | --- | --- | --- | --- |
| Item 1 | As the time of childbirth approaches, my worries increase. | 随着预产期临近，我对伴侣能否安全分娩的担忧不断增加 | As the due date approached, the worries about whether my spouse could deliver safely continued to grow. | Retained |
| Item 2 | During my spouse’s childbirth, I will feel helpless. | 当想到伴侣将要经历分娩，我就会感到无助 | When I think of that my spouse will go through childbirth, I feel helpless. | Retained |
| Item 3 | I worry about the quality of sex with my spouse after childbirth. | 我担心性生活质量在产后下降 | I worry that the quality of sex with my spouse will decline after childbirth. | Deleted after exploratory factor analysis. |
| Item 4 | During my spouse’s childbirth, I will feel restless. | 当想到伴侣将要经历分娩，我就会感到不安 | When I think of that my spouse will go through childbirth, I feel restless. | Retained |
| Item 5 | Because of my spouse’s fear of childbirth, I feel fear. | 由于伴侣对分娩的恐惧，让我也感到了恐惧 | My spouse's fear of childbirth make me feel fearful too. | Retained |
| Item 6 | I am afraid that I am not capable enough to support my spouse during childbirth. | 我害怕以我的能力，无法在分娩过程中给予伴侣足够的支持 | I am afraid that I am not capable enough to give my spouse enough support during childbirth. | Retained |
| Item 7 | I’m afraid that my spouse’s childbirth will be risky. | 我害怕伴侣分娩时出现意外 | I am afraid that my spouse will have an accident during childbirth. | Retained |
| Item 8 | I am afraid that dangerous medical interventions will be needed during childbirth. | 我害怕伴侣分娩过程中需要借助额外的医疗操作 | I am afraid that extra medical interventions will be needed during childbirth. | Retained |
| Item 9 | I will feel fear because of my spouse’s pain. | 我担心伴侣分娩时疼痛太剧烈，导致她难以忍受 | I am worried that the pain during delivery will be too intense for my spouse to bear. | Retained |
| Item 10 | I am afraid that my spouse’s health will be endangered due to childbirth. | 我害怕分娩会影响伴侣的健康 | I am afraid that my spouse’s health will be endangered due to childbirth. | Retained |
| Item 11 | I am afraid that my child’s health will be endangered due to childbirth. | 我害怕孩子在分娩时出现意外 | I am afraid that my baby will have an accident during childbirth. | Retained |
| Item 12 | During my spouse’s childbirth, I will feel fear. | 当想到伴侣将要经历分娩，我就会感到害怕 | When I think of that my spouse will go through childbirth, I feel fear. | Retained |
| Item 13 | I am afraid that the hospital staff will not take enough care of my spouse. | 我害怕伴侣得不到医护人员良好的照顾 | I am worry that the hospital staff will not take enough care of my spouse. | Retained |
| Item 14 | I’m afraid the hospital staff won’t treat me and my spouse respectfully. | 我害怕医护人员不会给予我和伴侣足够的尊重 | I am afraid that the medical staff will not treat me and my spouse with enough respect. | Retained |
| Item 15 | I am afraid that the hospital staff will not have enough skills to perform a safe childbirth. | 我害怕由于医护人员的技术不精或疏忽大意，导致伴侣无法安全分娩 | I am afraid that the hospital staff will not have enough skills or being careless to perform a safe childbirth. | Retained |
| Item 16 | I am afraid that the hospital will not have enough facilities and equipment for a safe childbirth. | 我害怕医院没有足够的设施和设备来保证安全分娩 | I am afraid that the hospital will not have enough facilities and equipment to ensure a safe delivery. | Retained |
| Item 17 | I am afraid that my child will be hospitalized in the neonatal intensive care unit after birth. | 我害怕孩子出生后需要进新生儿重症监护室 | I am afraid that my baby will be hospitalized in the neonatal intensive care unit after birth. | Retained |
| FFCS, the fathers’ fear of childbirth scale; C-FFCS-FHP, the final harmonized preliminary Chinese version of the FFCS; C-FFCS, Chinese version of the FFCS.  Note: The final C-FFCS is a 16-item and 3-dimension scale. Dimension 1 is named "fear of the health and safety of mother and baby," which includes items 1, 7, 8, 9, 10, 11, and 17; dimension 2 is named "fear of the quality of medical care services," which includes items 13, 14, 15, and 16; and dimension 3 is named "fear induced by individual factors," which includes items 2, 4, 5, 6, and 12. | | | | |
